# p73 as a Tissue Architect

**DOI:** 10.3389/fcell.2021.716957

**Published:** 2021-07-23

**Authors:** Laura Maeso-Alonso, Lorena López-Ferreras, Margarita M. Marques, Maria C. Marin

**Affiliations:** ^1^Departamento de Biología Molecular, Instituto de Biomedicina (IBIOMED), University of León, León, Spain; ^2^Departamento de Producción Animal, Instituto de Desarrollo Ganadero y Sanidad Animal, University of León, León, Spain

**Keywords:** p53-family, p73, tissue architecture, cell adhesion, actin cytoskeleton, cell polarity, central nervous system development, neurogenic niche

## Abstract

The *TP73* gene belongs to the p53 family comprised by p53, p63, and p73. In response to physiological and pathological signals these transcription factors regulate multiple molecular pathways which merge in an ensemble of interconnected networks, in which the control of cell proliferation and cell death occupies a prominent position. However, the complex phenotype of the *Trp73* deficient mice has revealed that the biological relevance of this gene does not exclusively rely on its growth suppression effects, but it is also intertwined with other fundamental roles governing different aspects of tissue physiology. p73 function is essential for the organization and homeostasis of different complex microenvironments, like the neurogenic niche, which supports the neural progenitor cells and the ependyma, the male and female reproductive organs, the respiratory epithelium or the vascular network. We propose that all these, apparently unrelated, developmental roles, have a common denominator: p73 function as a tissue architect. Tissue architecture is defined by the nature and the integrity of its cellular and extracellular compartments, and it is based on proper adhesive cell-cell and cell-extracellular matrix interactions as well as the establishment of cellular polarity. In this work, we will review the current understanding of p73 role as a neurogenic niche architect through the regulation of cell adhesion, cytoskeleton dynamics and Planar Cell Polarity, and give a general overview of TAp73 as a hub modulator of these functions, whose alteration could impinge in many of the *Trp73*^–/–^ phenotypes.

## Introduction

The *TP73* gene belongs to an evolutionary conserved family of transcription factors, the p53 family, with key functions to vertebrate’s biology. The genes that constitute this family, *TP53, TP63*, and *TP73*, have evolved from a common ancestor and, consequently, share a similar modular structure which consists of an amino-terminal transactivation domain (TAD), a central DNA binding domain (DBD) and a carboxy-terminal oligomerization domain (OD) (Dötsch et al., 2010). Although *TP53* was the first member of the family to be discovered (Lane and Crawford, 1979; Levine, 2020), *TP63* and *TP73* are the evolutionary older homologs (Kaghad et al., 1997; Yang et al., 1998; Belyi et al., 2010; Chillemi et al., 2017) and differ from *TP53* in that the full-length proteins that they encode contain a carboxy-terminal sterile a-motif (SAM) domain. This C-terminal region, involved in protein–protein interaction, might give p63 and p73 their unique signaling network of regulators and transcriptional targets (Serber et al., 2002; Straub et al., 2010). In addition, due to alternative splicing of the N-terminal and C-terminal regions and to the use of cryptic promoters, the *TP73* and *TP63* genes can be expressed as transcriptionally competent TA-isoforms or as N-terminally deleted DN-isoforms. Moreover, multiple alternative splicing at the 3′ region of the pre-RNA can give rise to C-terminal isoforms which, in the case of p63 and p73, can include the SAM domain (Vikhreva et al., 2018).

The first studied function of p73 was its p53-like growth suppressor capacity (Jost et al., 1997). Even though p53 is the central regulator of the cellular genomic integrity, TAp73 isoforms can perform similar functions in response to stress. Following DNA damage, TAp73 generates a coordinated response that induces either cell cycle arrest and DNA repair mechanisms, or provokes cell elimination signals leading to apoptosis or senescence (Pflaum et al., 2014). These p53-like responses, executed through the activation of target genes shared with p53, are known as p73-canonical functions. However, elimination of these canonical functions could not account for all the phenotypes observed in the knockout mice lacking all p73 isoforms, the *Trp73*^–/–^ (Yang et al., 2000). These animals display multiple maladies, including gastrointestinal and cranial hemorrhages, rhinitis, hippocampus dysgenesis and enlarged ventricles, female and male infertility, chronic infection and inflammation in lungs, sinus, and ears, and runting (Yang et al., 2000). Several laboratories, including ours, have demonstrated that the biological relevance of p73 does not exclusively rely on its growth suppression effects (Pflaum et al., 2014), but also on p73-non-canonical functions. Some of these functions, like the regulation of cell adhesion establishment, cytoskeleton dynamics, multiciliogenesis and Planar Cell Polarity (PCP) are related to the maintenance of the structural organization and homeostasis of different complex microenvironments, like the neurogenic niche and the ependymal barrier in the central nervous system (CNS), the respiratory and reproductive epithelia, or the vascular network. Thus, important questions arise: How does p73 orchestrate such an ample array of biological processes? Are there some common molecular functions underlying these phenotypes? May this p73 fundamental role be related to the organization of epithelia, a hallmark tissue of metazoans? Could this function represent a primitive p53/p63/p73-ancestor ability kept by p73 throughout evolution, and which is now fundamental in mammals?

## Diversifying Biological Activities: the Ying-Yang Mode of Action of p73 Isoforms

As mentioned before, the *Trp73* gene gives raise to functionally different TA and DNp73 isoforms (Candi et al., 2014). TAp73 proteins can transactivate canonical-p53 targets as well as non-p53 related genes involved in development and/or other cell growth associated functions (Engelmann et al., 2015; Wang et al., 2020). TA-isoforms differ in their transactivation efficiency and target gene specificity depending on their carboxy terminus (De Laurenzi et al., 1998; Ueda et al., 1999). Thus, TAp73 function will vary in a cell-context dependent manner and greatly depending upon their C-terminal domain (Logotheti et al., 2013; Vikhreva et al., 2018). Conversely, DN-isoforms can act as dominant-negative inhibitors of p53 and TAp73 and thus, have oncogenic properties (Ishimoto et al., 2002; Engelmann and Pützer, 2014), but they also carry out their own distinct p53/TAp73-independent transcriptional activities (Marqués-García et al., 2009; Wetterskog et al., 2009; Niemantsverdriet et al., 2012). The generation of transactivation-deficient DN-isoforms from the *TP73* gene is quite complex and has been reviewed elsewhere (Murray-Zmijewski et al., 2006; Engelmann et al., 2015). Briefly, there are two types of DN-p73 isoforms, the ones that originate from differential splicing events at the 5′-end of P1-derived transcripts (ΔEx2p73, ΔEx2/3p73, ΔN′p73; generally called ΔTA), and DN-isoforms, *per se*, which arise from the alternative P2 promoter within intron 3 (Stiewe et al., 2002; Buhlmann and Pützer, 2008; Engelmann et al., 2015). Even though most DN-isoforms are transcriptionally inactive, there are reports indicating that the 13 unique residues of DNp73 β and γ, together with the N-terminal PXXP motifs, constitute a novel activation domain capable of inducing some p53 target genes (Liu et al., 2004).

A detailed analysis of the total *Trp73*^–/–^ mice revealed a wide range of novel p73 physiological roles governing different aspects of cell and tissue physiology. However, p73 bimodal function has difficulted the identification of the responsible isoform for each of the observed phenotypes. The generation of isoform-specific knockout mice has provided a useful tool to disentangle some of the p73 isoforms-specific activities in various tissues and cellular processes, endorsing the proposed isoform-based model of p73 function.

Beginning with p73 tumor suppressor function, TAp73 deficient mice revealed an increased predisposition to spontaneous tumorigenesis (Tomasini et al., 2008), demonstrating the role of TAp73 as a tumor suppressor and substantiating previous reports of enhanced rate of spontaneous tumors in *Trp73* ± mice (Flores et al., 2005). On the other hand, elimination of DNp73 greatly inhibits tumor-forming capacity *in vivo* (Wilhelm et al., 2010). In this Ying-Yang model, while DNp73 possesses oncogenic properties that include impairment of the DNA damage-response pathway, cellular immortalization, as well as a dominant negative function of the p53/TAp73-canonical functions (Petrenko et al., 2003; Wilhelm et al., 2010; Billant et al., 2016), TAp73 tumor suppressor activity mainly relies on p53-canonical functions, like its ability to induce cell cycle arrest, apoptosis or regulation of DNA damage response, as well as other functions like immune cell regulation (Tomasini et al., 2008; Costanzo et al., 2014; Wolfsberger et al., 2021). It is noteworthy that TAp73, unexpectedly, activates anabolic pathways compatible with proliferation and promotion of cancer cells by regulating glucose metabolism to control cellular biosynthetic pathways and antioxidant capacity (Du et al., 2013; Fets and Anastasiou, 2013; Amelio et al., 2014). However, whether this metabolic effect reflects cancer-associated metabolic changes, or instead suggests a role for TAp73 in promoting adaptative cellular mechanisms to stress conditions (Agostini et al., 2014; Marini et al., 2018), remains to be determined and has been reviewed elsewhere (Nemajerova et al., 2018). Nevertheless, the lack of these p73-associated functions could not explain many of the cytoarchitecture alterations resulting from p73 deficiency *in vivo.*

In morphologically complex animals such as mammals, the establishment and maintenance of tissue structure and function, known as tissular architecture (Hagios et al., 1998), regulates the development and functionality of organs such as the digestive, respiratory, reproductive, neural, sensory, and vascular systems (Rodriguez-Boulan and Macara, 2014). Consequently, it would be expected that the disruption of a gene involved in tissue architecture could result in a plethora of developmental defects, such as the ones observed in the *Trp73*^–/–^ mice. Thus, we propose that some of the, apparently unrelated, phenotypes showed by these mice could reflect p73 requirement in the maintenance of functional tissue organization and we ask whether this task could represent one of the original roles of the p53/p63/p73-ancestor.

## Evolution of the p53 Gene Family and the Emergence of Tissular Architecture

**FIGURE 1:**
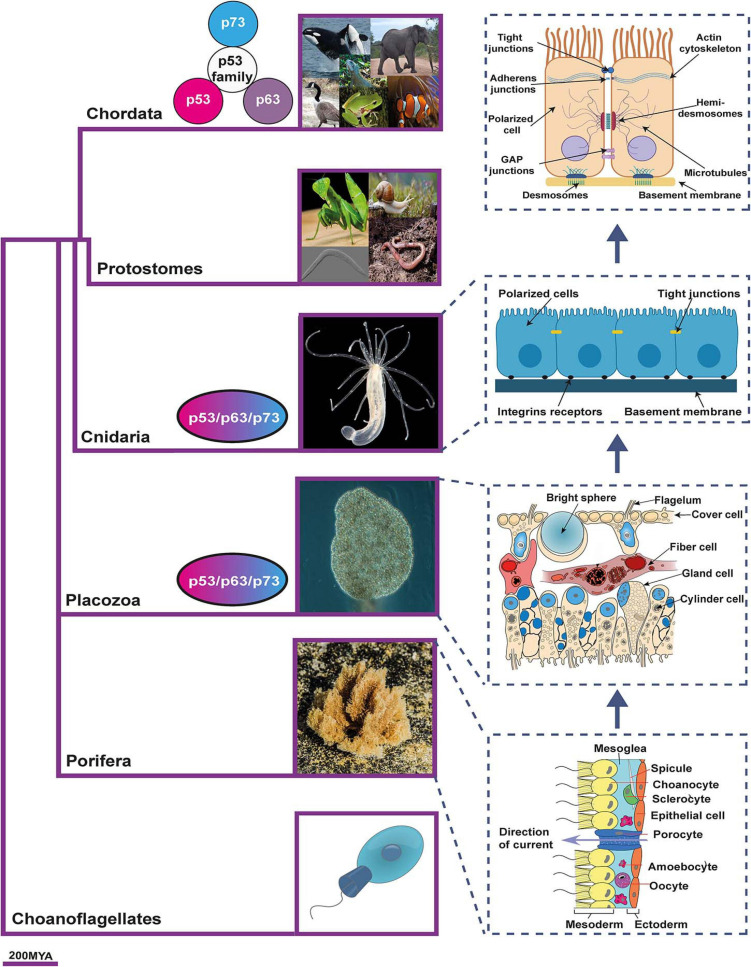
The emergence of epithelia and the proposed relationship with p53 family members phylogeny. p53/p63/p73-ancestor proteins appear for the first time in Placozoan and Cnidaria. Coincidentally, these organisms are the first ones to fulfill the three criteria that distinguish the “true” epithelial phenotype: i) cells displaying aligned polarity; ii) cells connected by belt-forming junctions; and iii) cells associated with extracellular matrix, with a basal lamina. As vertebrates develop, the p53/p63/p73-ancestor gave rise to the three members of the p53 family. The phylogenetic tree is based on Timetree public knowledge-base. The pictures were created with BioRender.com. Photos were a courtesy of Robert Aguilar, Smithsonian Environmental Research Center, United States (https://commons.wikimedia.org/wiki/File:Nematostella_vectensis_(I1419)_999_(30695685804).jpg) and Bernd Schierwater, Institute of Animal Ecology and Cell Biology, Hannover (Germany). https://commons.wikimedia.org/wiki/File:Trichoplax_adhaerens_photograph.png.

Identifying the original p53/p63/p73-ancestor functions might be elusive since data on the molecular characterization or function of most of these proteins are lacking. However, a canvass of the published data regarding consensus phylogenetic trees, together with the evidence of the p53 family presence spanning the early Metazoa through the primates, can lead to propose that the organization of the epithelia could be a primitive p73-function, since the appearance of the ancestral p73 paralogs seems to coincide with the phylogenetic emergence and organization of the epithelia (Belyi et al., 2010; Rutkowski et al., 2010; Åberg et al., 2017; Figure 1).

Within the animal kingdom, p53-family sequences are encoded in almost all sequenced genomes. The most primitive multicellular organisms encoding p53/p63/p73-ancestor-like proteins are the cnidaria starlet sea anemone *Nematostella vectensis*, and the placozoa *Trichoplax adherens* (Rutkowski et al., 2010). In these basal animals with radial symmetry, the ancestral gene is most closely related to a combined p63/p73-like gene (Belyi et al., 2010), and one or more ancestor sequences are found, while the radiation into p53, p63, and p73 protein coding genes has been described as a vertebrate event (Rutkowski et al., 2010; Figure 1).

It is precisely in placozoa where the p53/p63/p73-ancestor’s TAD first appeared and thus, the transactivation function (Åberg et al., 2017). Based on the presence of the conserved SAM domain and the greater sequence similarity between the vertebrate p63 and invertebrate p53/p63/p73-ancestor, an initial study suggested that the ancestral and invertebrate function of p53/p63/p73 mainly resembled the p63 vertebrate function (Rutkowski et al., 2010). However, a subsequent detailed phylogenetic analysis with a particular focus on the TAD led to the hypothesis that, since all three family members are equally evolutionarily close to the p53/p63/p73-ancestor, some of its primitive functions would be similar to that of p63, while others would resemble typical p53-functions and still others, not yet identified, could be p73-related functions (Åberg et al., 2017). So, which are these p73-related functions?

In *N. vectensis*, an invertebrate model susceptible to genetic analysis, it was shown that the p53/p63/p73-ancestor gene responds to DNA damage, causing apoptosis in its gametes (Pankow and Bamberger, 2007). These experiments prompted the idea that one of the functions of the p53/p63/p73-ancestor could be to trigger apoptosis in response to DNA damage to eliminate damaged germ cells (Pankow and Bamberger, 2007). This role, preserving genome stability of female germ cells, has been kept in mammals by p63 (Suh et al., 2006; Tomasini et al., 2008; Deutsch et al., 2011) where it serves as a quality control (QC) factor that ensures elimination of damaged oocytes before they can be recruited for ovulation (Suh et al., 2006; Livera et al., 2008). This QC function probably evolved into p53 tumor suppression function when more complex organisms required preservation of the somatic cells genome to prevent cancer (Levine, 2020).

Interestingly, p73, which is also involve in orchestrating germ cell maintenance, appears to exert this function not only through a QC mechanism, but also through the maintenance of the cytoarchitecture that provides the nurturing environment required during spermatogenesis (Holembowski et al., 2014; Inoue et al., 2014) and during the ovarian follicle development (Santos Guasch et al., 2018). This is in accordance with the idea that the regulation of tissue architecture could be one of the functions of the p53/p63/p73-ancestor that has been kept in vertebrate-p73. Nevertheless, whether p53/p63/p73-ancestor is required for epithelial organization in *N. vectensis*, or if the knockdown of the protein would result in defects in germ line maturation of this organism, remains unknown and further functional experiments are required.

The p53/p63/p73-ancestor role as a tissue organizer is supported by its apparent coincidental emergence with the primitive “true” epithelium, which first evolved in Placozoan and Cnidaria (Cereijido et al., 2004; Srivastava et al., 2008; Adams et al., 2010; Figure 1). True occluding epithelia are defined by cells that display an aligned polarity, are connected by belt-forming junctions that anchor the cytoskeleton and are associated with extracellular matrix (ECM) basal lamina (Fahey and Degnan, 2010). The placozoa *Trichoplax adhaerens*, which encodes the p53/p63/p73-ancestor like protein, is considered to have true occluding epithelia (Srivastava et al., 2008; Adams et al., 2010). It has an asymmetric epithelial bilayer with cells joined by apical junctions that manifest features of the Eumetazoa’s epithelia (Smith and Mayorova, 2019). In addition, its genome also encodes cell-surface adhesion proteins, all polarity complex members, a diverse set of genes that code for putative ECM proteins, as well as cytoskeleton linker proteins (Srivastava et al., 2008; Belahbib et al., 2018). Moreover, the ZO genes, which encode the ZO1-3 scaffold proteins of the tight junction, surge in Placozoa and are expanded in the Craniata (González-Mariscal et al., 2011).

The epithelium constitutes the core tissues of all metazoans, and it is the fundamental building block of all animal’s body structural design and function (Miller et al., 2013). The establishment and maintenance of tissular architecture requires the correct arrangement of the epithelial cells maintaining their central features: apico-basal cell polarity, cell-cell junctions and basal lamina, as well as their associated signaling complexes. Hence, architecture depends upon the organization of cell adhesion complexes, which hold epithelial cells together and connect them with the environment, as well as on the establishment and maintenance of an epithelial polarity program, including cellular cytoskeleton polarity. All these processes have been associated to p73 function in a variety of *in vitro* and *in vivo* models and could constitute the groundwork for its role as tissue organizer in several microenvironments (Zhang et al., 2012; Medina-Bolívar et al., 2014; Gonzalez-Cano et al., 2016; Fuertes-Alvarez et al., 2018; Santos Guasch et al., 2018). In this work, we will review the current understanding of p73 role as a brain architect. In particular, we will focus on the architecture of the subventricular neurogenic niche, which is of crucial importance for the maintenance of neural stem cell identity and for their neurogenic potential (Morante-Redolat and Porlan, 2019).

## p73 Fundamental Role in Mouse Brain Development

The role of p73 in the development of the CNS was recognized early on based on the profound defects of the total *Trp73*^–/–^ mice (Yang et al., 2000). These animals suffer from severe progressive *ex vacuo* hydrocephalus, hippocampal dysgenesis with abnormalities in the pyramidal cell layers (CA1 and CA3) and in the dentate gyrus, and loss of Cajal-Retzius (CR) neurons (Killick et al., 2011). However, the distinct elimination in the isoform-specific knockouts (TAp73KO and DNp73KO), generates subtle effects, and some of the phenotypes detected in the *Trp73*^–/–^ mice do not even appear in them (Tomasini et al., 2008; Tissir et al., 2009; Wilhelm et al., 2010). This is likely the reflection of either compensatory or redundant mechanisms in the absence of one of the isoforms, and/or possible differences in the genetic background of the mice models (Murray-Zmijewski et al., 2006). This, together with the ability of some isoforms to interact and regulate each other (Murray-Zmijewski et al., 2006), makes the study of the biological functions of this gene extremely complicated.

TAp73 is the predominant isoform expressed in embryonic neural stem cells (NSCs) (Tissir et al., 2009; Agostini et al., 2010; Gonzalez-Cano et al., 2010) and has been shown to regulate NSCs stemness and differentiation *in vitro* (Hooper et al., 2006; Agostini et al., 2010; Fujitani et al., 2010; Gonzalez-Cano et al., 2010; Talos et al., 2010). In accordance with TAp73 predominant role in neurogenesis, TAp73KO mice show hippocampal dysgenesis, but not ventricle enlargement or hydrocephalus (Tomasini et al., 2008). On the other hand, the DNp73KO mice display signs of neurodegeneration and a small reduction in cortical thickness and neuron number in older mice but do not show hippocampal abnormalities nor hydrocephalus (Tissir et al., 2009; Wilhelm et al., 2010). This led to propose that while DNp73 carries out neural protection functions, TAp73-isoforms are the main contributor to the development of the CNS. A recently developed mice model, with a selective knockout of the C-terminus of the full-length alpha isoform (*Trp73*Δ13/Δ13 mice), has shed new light on the role of p73 isoforms in the development of the murine brain (Amelio et al., 2020). These mice, which express the TAp73 beta isoforms at physiological levels, but lack the alfa-isoforms, suffer from a depletion of CR neurons in embryonic stages, leading to aberrant hippocampal architecture, reduced synaptic functionality and impaired learning and memory capabilities, altogether resembling the *Trp73*^–/–^ mice phenotype (Amelio et al., 2020). The authors concluded that the hippocampal dysgenesis was a consequence of deprivation of the CR cells, whose early function is the secretion of reelin that will orchestrate the arrival, size and stratification of all pyramidal neurons of the neocortex gray matter (Marín-Padilla, 2015). Interestingly, several groups have reported a link between cell adhesion and reelin-induced functions in corticogenesis (Sanada et al., 2004; Soriano and del Río, 2005; Sekine et al., 2012; Matsunaga et al., 2017). In the subventricular zone (SVZ), reelin controls the behavior of SVZ-derived migrating neurons, triggering them to leave prematurely the rostral migratory stream (Pujadas et al., 2010; Courtès et al., 2011). However, could lack of CR cells alone explain the severe structural defects of the SVZ in p73-deficient mice?

## p73 as an Architect of the SVZ and the Ependyma

Neurogenesis in the mammalian brain is complex and requires specialized microenvironments called “niches” (Scadden, 2006). In the adult brain, two niches haven been identified, the subventricular zone of the lateral walls of the ventricles (Alvarez-Buylla and Garcìa-Verdugo, 2002) and the subgranular zone of the dentate gyrus of the hippocampus (Doetsch et al., 1997). In the SVZ ventricular surface, multiciliated ependymal cells (EpCs) surround monociliated NSCs (B1 cells) forming a unique pinwheel architecture that is essential to maintain neurogenesis (Kuo et al., 2006; Mirzadeh et al., 2010; Paez-Gonzalez et al., 2011). In these pinwheels, the EpCs are polarized within the plane of the tissue, a process that is known as PCP. This essential feature of animal tissues (Butler and Wallingford, 2017) makes feasible that EpCs coordinate cilia beating and direct the cerebrospinal fluid circulation; therefore, PCP disruption results in ciliopathies and hydrocephalus (Marques et al., 2019).

**FIGURE 2:**
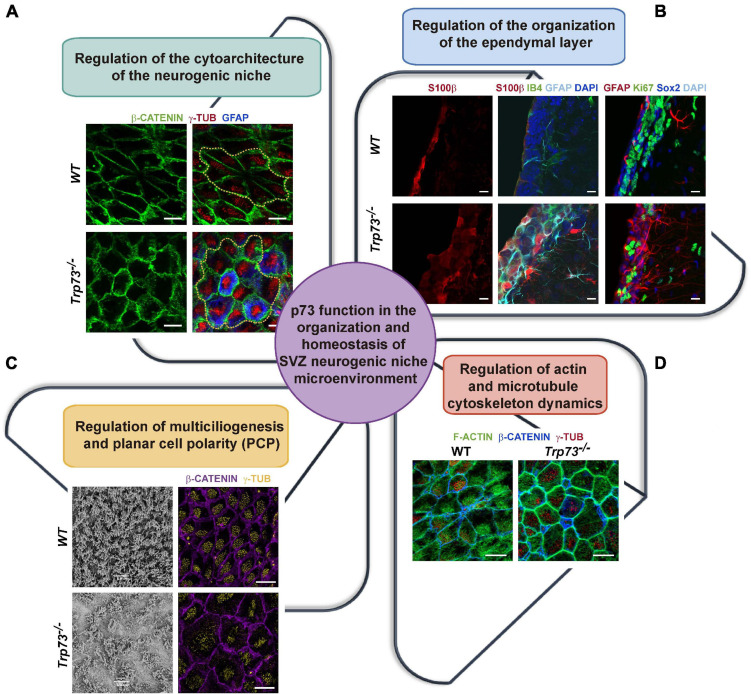
p73 role as an architect of the SVZ neurogenic niche. **(A)** p73 is essential for the formation of the neurogenic pinwheels. In the absence of p73, EpCs show an aberrant membrane morphology and fail to organize into pinwheels, disrupting SVZ niche cytoarchitecture. Analysis of lateral ventricle wall whole-mounts of the indicated genotypes at postnatal day P160 immunostained for b-catenin (green), g-tubulin (red), and GFAP (blue). Pinwheel structures are marked by dotted yellow lines. Scale bars: 10 mm. **(B)** p73-deficient EpCs cell-junction defects compromise the integrity of the ependymal layer and halt the formation of the mono-stratified epithelium. In addition, cells with abnormal marker expression profiles are observed in p73KO brains. Coronal sections of the lateral wall of the lateral ventricle from P15 WT and p73KO mice were stained with the indicated antibodies and analyzed by confocal microscopy. Scale bars: 10 mm. **(C)** p73 is required for ciliogenesis and planar cell polarity establishment. p73KO cells displayed an abnormal cilia organization and basal body-cluster displacement. SEM (P7 mice) and confocal microscopy analysis (P15 mice, γ-tubulin, yellow; β-catenin, purple) of WT and p73KO lateral ventricle wall wholemounts. **(D)** p73 is also necessary for the formation of the polarized apical and sub-apical actin lattices in EpCs. Confocal images of WT and p73KO P15 wholemounts displaying as indicated: actin cytoskeleton (phalloidin, green), basal bodies (γ-tubulin, red) and the cell membrane (β-catenin, blue). Images from Dr. Marin’s research group (Gonzalez-Cano et al., 2016, Fuertes-Alvarez et al., 2018).

One of the most striking features of the *Trp73*^–/–^ mice is the complete lack of cytoarchitecture in the SVZ neurogenic niche (Gonzalez-Cano et al., 2016; Fuertes-Alvarez et al., 2018; Marques et al., 2019). TAp73, but not DNp73, is expressed in the EpCs and its precursors, the radial glia cells (Tissir et al., 2009; Hernández-Acosta et al., 2011; Medina-Bolívar et al., 2014; Fujitani et al., 2017), and it is essential for the ependymal cell assembly into neurogenic pinwheels (Gonzalez-Cano et al., 2016). Total p73 deficiency results in altered pinwheels where the EpCs have an aberrant membrane morphology with waves and pleats (Figure 2A), reflecting severe defects on the intercellular junctions at the apical surface of these cells (Gonzalez-Cano et al., 2016). These cell-junction defects also compromise the integrity of the ependymal barrier (Figure 2B), all pointing toward a TAp73 role in the establishment of intercellular junctions.

Compiled evidence indicates that the combination of alterations in vesicle trafficking, cell junction defects and loss of ependymal barrier integrity constructs a common pathway leading to ventricular zone disruption (Ferland et al., 2009). All these processes, as we will discuss later on, have been associated to TAp73 transcriptional regulation. Moreover, it is now accepted that abnormal junction complexes in the cells of the ventricular zone, including NPC, may lead to disruption of the ventricular and subventricular zones, resulting in hydrocephalus and abnormal neurogenesis (Rodríguez et al., 2012; Guerra et al., 2015). Thus, the “cell junction pathology,” resulting from p73 ablation, might be underneath some of the functional and structural alterations of the *Trp73*^–/–^ mice in the CNS, but also in other organs.

*Trp73* function in the establishment of intercellular junctions has been strongly demonstrated in the reproductive epithelia. In the multilayered epithelia of the seminiferous tubules, lack of total p73 or TAp73 results in defective cell-cell adhesion of germ cells with Sertoli cells, leading to the premature detachment of the developing spermatids and concomitant cell death (Holembowski et al., 2014; Inoue et al., 2014). Interestingly, Sertoli cells do not express p73, but they are also affected by the loss of germ cell adhesion in *Trp73*^–/–^ testes, losing their characteristic morphology as well as the inter-Sertoli cell adhesions that form the blood-testis barrier (Holembowski et al., 2014). Furthermore, in the developing ovary, p73 regulates a set of core genes involved in biological adhesion, thus acting as a regulator of intercellular adhesion, ECM interactions, and cell migration processes required for proper follicle development (Santos Guasch et al., 2018).

However, there are other pathological features of the *Trp73*^–/–^ mice in which the possible link with cell junctional defects has not been addressed. That is the case of the chronic respiratory and gastrointestinal infections that these animals suffer from Yang et al. (2000). While p73 signaling has been associated to the epithelial cell response to infections caused by, for example, *H. pylori* (Wei et al., 2008), the cause of the increased susceptibility to infections, *per se*, in p73-deficient animals is not understood. Interestingly, loss of epithelial integrity has been widely demonstrated to be central to pathogen infection, since disruption of junctional integrity facilitates viral or bacterial entry and spread (Lu et al., 2014). Thus, it would be interesting to address whether the aforementioned “cell junction pathology” resulting from p73-deficiency is at the root of the susceptibility to chronic infections in these mice.

Another interesting scenario are the defects in the vascular network described in the *Trp73*^–/–^ mice. These mice exhibit extensive gastrointestinal and cranial hemorrhages (Yang et al., 2000) which are suggestive of vascular fragility or other defects in their vascular compartment. Our group reported that *Trp73* deficiency *in vivo* results in aberrant retinal vascular morphology, while *in vitro* ablation of p73 in 3D mESC and iPSC models impairs the early stages of vasculogenesis, demonstrating the essential role of *Trp73* in vascular development (Fernandez-Alonso et al., 2015). Compiled data from several groups supports the idea that this function is, at least in part, due to DNp73 modulation of pro-angiogenic signaling pathways (Dulloo et al., 2015a; Fernandez-Alonso et al., 2015; Stantic et al., 2015). As for TAp73, its role in vascular morphogenesis is unclear, especially regarding tumor angiogenesis. Collectively, several studies have demonstrated that TAp73 can act as both a positive and negative regulator of tumor angiogenesis under different spatio-temporal contexts and therefore, a bi-functional role for TAp73 in angiogenesis has been proposed (Amelio et al., 2015; Dulloo et al., 2015b; Stantic et al., 2015, reviewed in Sabapathy, 2015). However, TAp73 physiological function in vascular morphogenesis still needs to be addressed. Regarding the latter, Stantic et al. reported that TAp73-deficient tumor cells produce and secrete factors that disrupt intercellular contacts in endothelial cells cultured with the tumor cells-conditioned media (Stantic et al., 2015). However, whether the absence of TAp73 in endothelial cells leads to junctional defects, *in vivo* and/or *in vitro*, and the possible consequences of this in vascular morphogenesis remains an important open question.

## p73 Regulation of Cytoskeleton Dynamics at the Center Stage of PCP and Multiciliogenesis Establishment

An in-depth analysis of the SVZ of *Trp7*3^–/–^, TAp73KO, and DNp73KO mice revealed that the lack of total p73 results in profound alterations of ependymal multiciliogenesis and PCP establishment (Gonzalez-Cano et al., 2016; Fuertes-Alvarez et al., 2018). The role of p73 on ciliogenesis is complex and has been reviewed elsewhere (Marques et al., 2019; Nemajerova and Moll, 2019). p73-deficiency affects different stages of the process depending on the absence of one or both isoforms. EpCs with total lack of p73 have severe ciliary defects, with many cells lacking ciliary axoneme and others displaying disorganized and aberrant cilia (Gonzalez-Cano et al., 2016; Figure 2C). TAp73 role in cilia formation has been demonstrated in other systems such as in the respiratory and reproductive epithelia, where TAp73 was found to function as a master transcriptional regulator governing motile multiciliogenesis (Marshall et al., 2016; Nemajerova et al., 2016).

TAp73 isoform elimination in TAp73KO mice does not recapitulate total *Trp73*^–/–^ phenotype in ependymal cells but rather results in a mild phenotype. In these mice, most EpCs display ciliary axoneme but with defective basal body docking and a “disheveled” appearance (Fuertes-Alvarez et al., 2018; Wildung et al., 2019). These defects are most likely due to the observed alterations -linked to TAp73 deficiency- in the sub-apical actin cytoskeleton dynamics and microtubule polarization, which regulates basal body docking and spacing (Vladar and Axelrod, 2008; Werner et al., 2011). On the other hand, DNp73-deficient EpCs do not display any ciliary defects indicating that, in the presence of TAp73, DNp73 is not necessary to orchestrate ciliogenesis. These data suggest that redundant ciliary programs are induced in the absence of TAp73 but that cannot compensate total p73 deficiency. In the same line, the *Trp73*Δ13/Δ13 mice do not display any apparent alteration in the airway ciliated epithelium, neither in the EpCs, suggesting that p73β or other redundant mechanisms can substitute the function of the longer isoform p73α (Buckley et al., 2020).

The spatial and temporal frame of TAp73 expression in the developing brain is an important question to pinpoint its physiological function. In mice, the transition of neuroepithelial cells to radial glial cells occurs between the embryonic days (E) 10 and 12, when the tight junctions that couple neuroepithelial cells convert into adherens junctions, and the cells acquire features associated with glial cells (Fuentealba et al., 2015). It is noteworthy that TAp73 expression in the *Trp73*Δ13/Δ13 mice was detected in the neuroepithelium from E11.5 to E16.5 (Amelio et al., 2020). This is an important stage during CNS development in mice, since birth dating experiments suggest that the majority of telencephalic EpC are produced between E14 and E16 (Spassky et al., 2005). By E16 the primary cilia of many transforming radial glial cells have become asymmetrically displaced within its apical surface, a key step in the ependymal cell’s differentiation and in the establishment of the organizations of the SVZ neurogenic niche (Redmond et al., 2019). Moreover, Fujitani and colleagues proposed that p73 regulates embryonic primary ciliogenesis, since disruption of p73 (both TA and DNp73) during early postnatal EpC development (P1-P5) did not cause hydrocephalus (Fujitani et al., 2017). Nevertheless, compiled data strongly support the idea that p73 functions at several stages during radial glial cell transformation into EpC (Marques et al., 2019). Thus, considering the reported early expression of TAp73 during development (Amelio et al., 2020), should we expect the cytoarchitecture of the SVZ in these mice to be maintained? or by the contrary, would sustained expression of TAp73 will be required for organization? Do these mice display PCP defects related to cell-junctions and cytoskeleton alterations as the TAp73KO mice do?

The coordinated polarization of EpC motile cilia within the plane of the tissue allows the synchronized beating that drives directional fluid flow and is required for EpC functionality (Ohata and Alvarez-Buylla, 2016). Multiciliated ependymal cells display two types of PCP, translational PCP (tPCP) and rotational (rPCP). While tPCP is unique to EpCs and is defined by the asymmetric localization of the cilia cluster at the anterior apical surface, rPCP refers to the unidirectional orientation of the motile cilia within the cell (Mirzadeh et al., 2010). PCP is established by asymmetric localization of PCP-core regulatory proteins complexes at opposite sides of the apical membrane (Boutin et al., 2014; Ohata et al., 2014) and it is driven by multiple global cues that guide the subcellular enrichment of PCP-core proteins such as Frizzled, Vangl, Celsr, Disheveled and Prickle (Butler and Wallingford, 2017). PCP-core components then self-assemble into mutually exclusive complexes at opposite sides of a cell to communicate polarity between neighboring cells and direct polarized cell behaviors. *Trp73* is necessary for the efficient establishment of both types of PCP (Gonzalez-Cano et al., 2016; Fujitani et al., 2017; Fuertes-Alvarez et al., 2018). In the absence of p73, or even TAp73, PCP-core complexes fail to assembly at opposite intercellular junctions of EpCs, and therefore, polarity is not established, suggesting that p73 might regulate early up-stream events of PCP establishment (Gonzalez-Cano et al., 2016; Fujitani et al., 2017; Fuertes-Alvarez et al., 2018).

But how do the cells, and for that matter TAp73, establish this asymmetry? Several processes have been involved in PCP-core complex’s asymmetry, from cilia-driven fluid flow to cellular rearrangements dependent on cytoskeletal polarity (Takagishi et al., 2017). It is important to bear in mind that asymmetry can be established independently of cilia, through the intrinsic chirality of the actomyosin cytoskeleton (Juan et al., 2018). Polarity in epithelial tissues is known to be influenced by cell-cell junctions, cytoskeletal elements, and by cell-cell signaling. Our group has demonstrated that p73 regulates PCP, at least in part, through TAp73-modulation of actin and microtubule dynamics (Fuertes-Alvarez et al., 2018). The actin cytoskeleton of multiciliated ependymal cells is organized into a cortical network, implicated in cell shape changes, and two interconnected apical and subapical networks that enclose the basal bodies contributing to their spacing and to the synchronization of cilia beating (Werner et al., 2011). p73 is required for the localization and organization of these actin networks, as p73-deficiency results in the complete lack of polarized apical and sub-apical lattices, in the formation of a thick actin cortex and the disposition stress fibers, all with a concurrent change in cell morphology (Fuertes-Alvarez et al., 2018; Figure 2D).

In recent years it has become apparent that actin-microtubule crosstalk is particularly important for the establishment of neuronal and epithelial cell shape and function (Dogterom and Koenderink, 2019). Microtubules crosstalk with PCP at two stages (Vladar et al., 2012; Werner and Mitchell, 2012; Takagishi et al., 2017). First, at the initial polarization establishment, when the microtubule-network grows asymmetrically from the center of the cell toward the anterior region of the apical cell cortex, contacting the plasma membrane at the intercellular microtubule-anchoring points which are polarized at tissue level. Second, when these polarized microtubules asymmetrically transport the PCP-core proteins to the correct anterior/posterior cell boundary (Shimada et al., 2006; Harumoto et al., 2010). Lack of p73 blunts the formation of polarized microtubule-anchoring points at cell junctions, suggesting that impairment of microtubule-dynamics is at the root of the defect in p73-deficient cells (Boutin et al., 2014; Takagishi et al., 2017).

As we will discuss below, the role of p73 as a regulator of cellular cytoskeleton dynamics has been shown in several systems. Thus, we should ask whether p73 regulation of PCP is a general function operating in various tissues and organs, or, on the contrary, it is limited to ependymal cells. Emerging data assign new roles for PCP in postnatal contexts, including formation of functional organs such as lungs and kidneys (Henderson et al., 2018), all highlighting the need of polarized cellular behaviors for proper development and function of diverse organs. In particular, asymmetric distribution of PCP-core complexes at intercellular junctions is required for the correct cilia orientation in other epithelia, like the trachea, oviduct and the organ of Corti. TAp73-deficiency results in ciliary defects in trachea and the oviduct in *Trp73*^–/–^ and TAp73KO mice (Marshall et al., 2016; Nemajerova et al., 2016). However, the planar organization of these epithelia has not been addressed. Furthermore, defects in PCP have been implicated in human pathologies, leading to the obvious and interesting question of whether alterations in p73 expression or mutations could be implicated in these diseases.

## TAp73 as a Central Hub That Modulates Transcriptional Programs Involved in Cytoskeleton Dynamics and Cellular Adhesion

The main question that arises is: how does TAp73 modulate all this variety of biological processes? The mechanism of TAp73 role in NSC stemness and neural differentiation is complex and relies on p73 regulation of different transcriptional profiles. In recent years, several genes involved in proliferation, differentiation and/or self-renewal of NSC, like *Sox-2, Hey-2, Trim32, and Notch*, have been postulated as TAp73 transcriptional targets (Hooper et al., 2006; Agostini et al., 2010; Fujitani et al., 2010; Gonzalez-Cano et al., 2010, 2016; Talos et al., 2010). TAp73 is also implicated in the regulation of post-mitotic neuron function by modulating the expression of p75NTR or GLS2, which are associated to axonal growth and dendritic arborization and neuronal metabolism, respectively (Niklison-Chirou et al., 2017). However, the profound structural alterations observed in the SVZ architecture of the *Trp73*^–/–^ mice cannot exclusively be explained by defects in cellular proliferation, differentiation, self-renewal or even metabolic defects. Regarding the regulation of multiciliogenesis, the compiled data identified over 100 putative p73 target genes that regulate multiciliated cell differentiation and homeostasis and revealed *Foxj1* as a direct TAp73 target, supporting a model in which p73 acts as a regulator of multiciliogenesis through direct and indirect regulation of key genes (Marshall et al., 2016; Nemajerova et al., 2016). TAp73 undoubtedly acts as a master regulator of ciliogenesis and *Trp73* total loss results in dramatic ciliary defects in EpCs, oviduct, middle ear and respiratory tract (Gonzalez-Cano et al., 2016; Marshall et al., 2016; Nemajerova et al., 2016; Fujitani et al., 2017). Still, the elimination of this ciliary function alone could not explain the abovementioned structural alterations. Interestingly, lack of TAp73 in EpCs results in defective actin and microtubule networks with a concomitant loss of PCP even though the ciliary axonemal growth remains unaffected, suggesting that TAp73 uncouples ciliogenesis from PCP establishment and regulates multiple independent, but interrelated, transcriptional programs to orchestrate these processes. In this regard, our group has demonstrated that mechanistically, TAp73 modulates actomyosin dynamics, at least in part by the transcriptional regulation of the myosin light chain kinase (MLCK), the activator of non-muscle myosin II (NMII) (Fuertes-Alvarez et al., 2018), which functions as a cortical organizer to concentrate E-cadherin to the *zonula adherens* (Smutny and Yap, 2010). TAp73 also activates transcriptional programs involved in the regulation of microtubule-dynamics and Golgi organization signaling pathways, both necessary for PCP establishment (Fuertes-Alvarez et al., 2018). Along the same lines, some of the genes significantly bound and regulated by p73 in multiciliated trachea cells, like *Traf3ip1* and *Tubb4b* (Marshall et al., 2016), are known to regulate the acetylation, polymerization and stabilization of microtubules (Berbari et al., 2011; Bizet et al., 2015; Sobierajska et al., 2019) or to be involved in vesicle trafficking, like *Sec24b*, that selectively sorts Vangl2 to regulate PCP (Merte et al., 2010).

A growing body of work indicates that the functional interaction between cell junctions and actin and microtubule cytoskeleton is critical for epithelial morphogenesis (Robinson, 2015; Adil et al., 2021). As discussed before, a possible common denominator to many of the p73-deficient phenotypes is the cell junctional defect and cytoskeleton dynamics alterations, suggesting a general function of TAp73 as a central hub that modulates transcriptional programs involved in these processes. To address whether this is the case, we revisited some of the transcriptomic studies that have been used to identify TAp73 target genes. We selected the genome-wide studies from Koeppel et al. (2011), Santos Guasch et al. (2018), and López-Ferreras et al. (2021). In their work, Koeppel et al. used the p53-deficient, TAp73β-inducible, osteosarcoma cell line Saos2-Tet-On to characterize the molecular basis for the different physiological functions of p73. In the second study, the authors measured global gene expression changes by RNA-seq after ectopic expression of TAp73β in mouse granulosa cells (MGCs) isolated from *Trp73*^–/–^ female mice. They express TAp73β basing their decision on previously published data showing that TAp73β exhibits the highest level of transcriptional activity among p73 isoforms (Lee and La Thangue, 1999; Ueda et al., 1999). Lastly, López-Ferreras et al. (2021) characterized the transcriptomic profile of E14TG2α mouse embryonic stem cells (mESCs) in which they specifically inactivated the TAp73-isoform (E14-TAp73KO) using the CRISPR/Cas9 system. In particular, we selected the analysis performed by the authors under differentiation conditions, since this approach offers the advantage of investigating p73 regulation in a physiological context that recapitulates early developmental stages where p53 family members are known to be upregulated (Medawar et al., 2008; Wang et al., 2017). Using the published RNA-seq data, we focus on the differentially expressed genes (DEGs) that were upregulated upon TAp73-expression in MGCs and Saos-2-Tet-On, or downregulated in E14-TAp73KO. To limit our analysis to genes that are potentially direct TAp73 targets, we compared those DEGs lists with a compilation of candidate genes with p73 genomic binding sites identified through ChIP-seq-studies (Koeppel et al., 2011; Marshall et al., 2016; Santos Guasch et al., 2018) and analyzed them with DAVID Bioinformatics Resources 6.8 (Huang et al., 2008, 2009) to identify enriched biological GO terms and obtain a functional annotation clustering.

**FIGURE 3:**
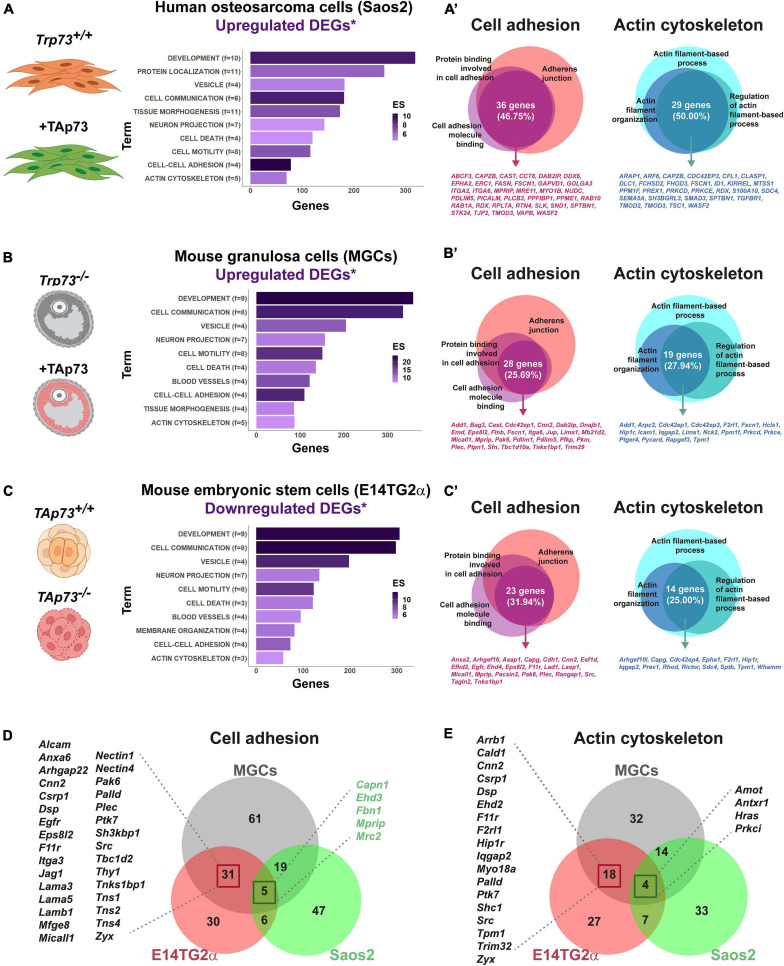
p73 is a central hub of cellular adhesion and cellular cytoskeleton dynamics. For the indicated cell models, putative TAp73 target genes (DEGs*) were obtained by comparison of differentially expressed genes (DEGs) derived from RNA-seq studies with candidate genes containing p73 binding peaks (*) according to ChIP-seq studies by Koeppel et al. (2011), Marshall et al. (2016), and Santos Guasch et al. (2018). A total number of 736 genes for Saos-2-Tet-On cells, 679 genes for MGCs, and 709 genes for E14-TAp73KO were analyzed with DAVID Bioinformatics Resources 6.8. Functional annotation clustering was performed and enriched biological GO terms for Saos2-Tet-On cells **(A)**, MGCs **(B)** and E14-TAp73KO **(C)** are represented. Overlapping genes within GO terms related to “Cell-cell adhesion” and “Actin cytoskeleton” were furthered identified and represented for the three cell models **(A’–C’)**. Comparison of the whole list of DEGs assigned to these clusters between the three cell models is shown **(D,E)**. Publicly available datasets were analyzed in this study and can be found here: GSE15780, PRJNA310161; PRJNA437755. The pictures were created with BioRender.com.

Highlighting the significance of p73 non-canonical functions, one of the clusters with the highest enrichment score for the three analyzed models was related to “Development” (GO:0048731∼system development, GO:0048513∼animal organ development, GO:0048869∼cellular developmental process, etc.), even ahead of p73 role in controlling cell death/cell proliferation (Figures 3A–C). Also consistent with the expected behavior for a tissular architect, functions related to “Cell-cell adhesion” and “Actin cytoskeleton” were significantly enriched in all the selected gene lists. The preservation of tissue function not only relies on biophysical cues, but also on the correct biochemical communication between cells and with the ECM to relay positional information. Accordingly, clusters like “Cell communication” and “Vesicular transport” (GO:1903561∼extracellular vesicle; GO:0070062∼extracellular exosome) showed a highly significant enrichment score. This coupling at molecular level between different aspects of tissue architecture reinforces p73 role as a key regulator of the organization and homeostasis of complex microenvironments. On a similar note, other annotation clusters like “Cell migration,” “Neuron projection,” or “Blood vessel development” were also highly significant. These findings are consistent with previous reports of p73 regulation of cell migration (Landré et al., 2016), and with the essential p73 function in neural and vascular development previously discussed in this review, altogether indicating that the cellular systems analyzed here are excellent models to identify and study putative TAp73 target genes.

To gain insight into p73 regulation of cell adhesion and actin cytoskeleton dynamics we explored, for each cell type, the overlapping genes within the main GO terms included in these clusters (Figures 3A‘–C’ and Supplementary Table 1). Regarding cell adhesion, we focused on p73 putative targets that were common to the terms “GO:0005912∼adherens junction,” “GO:0050839∼cell adhesion molecule binding” and “GO:0098631∼protein binding involved in cell adhesion.” Within these, we found: (i) genes encoding integrins such as Itga2 and Itga6, or the *Zonula occludens* protein ZO2, a scaffold protein that physically links transmembrane tight junction proteins to the apical cytoskeleton of actomyosin (Raya-Sandino et al., 2017), in Saos2 cells; (ii) the LIM domain and actin binding 1 protein LIMA1, a demonstrated direct transcriptional target of TAp73 whose activity is counteracted by DNp73 (Steder et al., 2013), or the cytoskeleton-related protein PDLIM5, also known as ENH (Enigma homolog) (Huang et al., 2020), in MGCs; and (iii) genes encoding E-cadherin or plectin-one of the major cytoskeletal linker proteins- (Wiche et al., 2015), in E14TG2α cells. Some of these DEGs were shared between cell models, although it should be noted that there was only one DEG associated to the analyzed GO terms that was common to the three cell types. This gene encoded the myosin phosphatase Rho-interacting protein MPRIP, a scaffold protein that associates with the actomyosin cytoskeleton, regulating myosin light chain phosphatase (MLCP), and that has been involved in the regulation of stress fibers (Koga and Ikebe, 2005). Whether this gene is a true p73 transcriptional target remains to be validated.

A similar situation occurred for genes related to actin cytoskeleton regulation. In this case, we draw our attention to the functional annotation terms “GO:0030029∼actin filament-based process,” “GO:0032970∼regulation of actin filament-based process” and “GO:0007015∼actin filament organization.” Among the DEGs shared within cell models, we could find some genes playing relevant roles for cytoskeleton dynamics, like *Fscn1* (DEG in Saos-TetOn and MGCs) or *Scd4* (DEG in Saos-TetOn and E14TG2α). FSCN1 is an actin binding-protein involved in the formation of essential cell structures for migration, cell-to-cell interactions and cell-matrix adhesion (Lamb and Tootle, 2020); therefore, different studies have highlighted its importance for tissue architecture, particularly when it is disrupted in tumor microenvironments (Liu et al., 2021). Syndecans are transmembrane proteins which act as communicators between intracellular, cell surface and ECM components (Elfenbein and Simons, 2013). Loss of Syn-4 alters the actin network and affects focal adhesions, decoupling vinculin from the actin filaments (Cavalheiro et al., 2017). Finding several genes related to cell-ECM interactions when collectively analyzing these transcriptomic studies may imply that the role of p73 as a tissue architect goes far beyond than anticipated and points to p73 involvement in integrin associated-signaling, as already suggested by Xie et al. (2018) or López-Ferreras et al. (2021).

The DEG analysis of cell adhesion and cytoskeleton dynamics clusters for the individual models led as to ask whether we could define a more global transcriptional profile by comparing the whole list of DEGs assigned to these clusters in Saos2-TetOn, MGCs, and mESCs (Figures 3D,E). For both biological functions, the cell models with a stronger epithelial component (MGCs and E14TG2α) shared a core set of genes regulated by p73 (36 genes for “Cell-cell adhesion” and 22 genes in the case of “Actin cytoskeleton”), supporting the existence of a “p73 gene signature” associated to tissue architecture. Interestingly, Koeppel et al. (2011) proposed that TAp73β seems to induce target genes that fall into KEGG functional categories linked to metastasis, such as focal adhesion, ECM–receptor interaction and actin cytoskeleton regulation. On the other hand, the p53-signaling pathway is the first functional category that appears in the KEGG pathway analysis for TAp73β, although biological process such as “cell adhesion” and “biological adhesion” were included in their GO functional analysis. In agreement, other GO studies demonstrate that overexpression of TAp73β can also regulate these functions in diverse cellular contexts (Fuertes-Alvarez et al., 2018; Santos Guasch et al., 2018; López-Ferreras et al., 2021). It is worth noting that the molecular networks involved in cell-to-cell adhesion revealed by Santos Guasch et al. (2018) in a total *Trp73* knockout scenario are further supported by the recent study of López-Ferreras et al. (2021), with specific inactivation of TAp73 in mESCs, emphasizing the interest of this fine-tuned cellular model to decipher the role of the *Trp73* gene isoforms. Altogether, the analyzed studies indicate that the integration of -omics data could be a very valuable strategy to provide a more comprehensive dissection of p73 regulated molecular networks and, overall, they place p73 as a central hub in the regulation of cell adhesion and cytoskeleton dynamics, two cornerstones for tissue architecture.

## Conclusion

*TP73* belongs to one of the most intensively studied gene families in molecular oncology. The considerable interest stems from the fact that most human tumors have subverted the function of the founding member of this family, the p53 protein. Thus, since its serendipitous discovery (Kaghad et al., 1997), p73 tumor suppression function was expected by virtue of its homology with p53 and its localization to chromosome 1p36, a region that is frequently deleted in a variety of tumors (Ichimiya et al., 1999). However, this function has been a matter of controversy, fueled by the fact that inactivation of the *TP73* gene is a very rare event in cancers involving chromosome 1p (Han et al., 1999; Inoue and Fry, 2014). Moreover, the observation that viral oncoproteins discriminate between p53 and p73 suggests that the functions of these two proteins may differ under physiological conditions (Marin et al., 1998).

**FIGURE 4:**
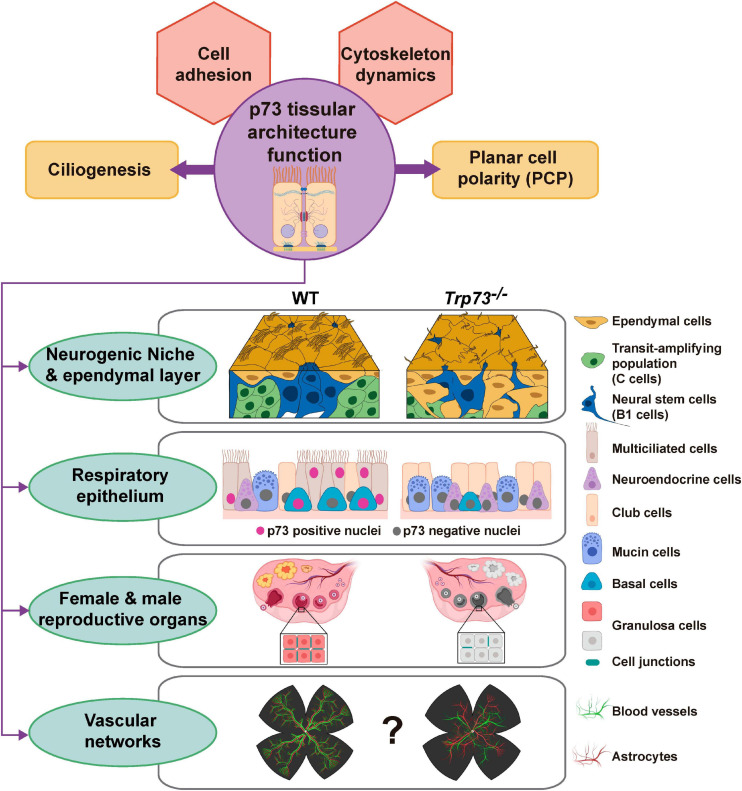
Overview of the proposed p73 novel role as a tissue architect. We present a model in which p73 would act as a tissue architect by regulating transcriptional hubs involved in cellular adhesion and cytoskeleton dynamics. In determined spatio-temporal contexts, TAp73 will interplay with other transcriptional programs to orchestrate morphogenic processes like ciliogenesis and/or PCP, ensuring the correct overall tissular architecture in complex microenvironments such as neurogenic niche, the respiratory and reproductive epithelium and, maybe, the vascular network. The pictures were created with BioRender.com.

The discovery of the TA- and DN-p73 isoforms with antagonist anti- and pro-oncogenic functions, and the TAp73KO mice predisposition to spontaneous tumorigenesis, demonstrated TAp73 role as a tumor suppressor gene (Tomasini et al., 2008). However, there is growing evidence indicating that while TAp73 has a role in tumor suppression, it is likely to be secondary (reviewed in Wang et al., 2020). The complex phenotype of the *Trp73* deficient mice have revealed that p73 function is essential for the organization and homeostasis of different complex microenvironments governing various aspects of tissue physiology. Altogether, this has raised the idea that TAp73 was not evolved for tumor suppression, but rather to perform unique functions in regulating developmental processes through p53-independent mechanisms (Wang et al., 2020). We propose that some of the, apparently unrelated, phenotypes observed in *Trp73*^–/–^ mice are the reflection of the p73 requirement for the establishment and/or maintenance of tissue organization (Figure 4). This function as a tissular architect might represent one of the original roles of the p53/p63/p73-ancestor. Furthermore, it is important to bear in mind that this function might reconnect with TAp73 tumor suppressor function, since a recent report proposes that the metastatic programs arise from the reactivation, outside of its homeostatic context, of normal embryonic developmental transcriptional modules (Logotheti et al., 2020). Thus, in a similar way, deregulation of p73 expression during tumor progression could result in alterations of the transcriptional nodes that p73 regulates as a tissue architect, playing a pivotal role during metastasis establishment.

In this model, p73 regulates distinct transcriptional nodes in a hierarchical manner that would functionally interact with each other in a cell context and time dependent manner. Cell adhesion mechanisms are responsible for assembling cells together and, along with their connections to the internal cytoskeleton, determine the overall architecture of the tissue (Gumbiner, 1996). In this way, TAp73-regulated transcriptional hubs, involved in cytoskeleton dynamics and cellular adhesion, will constitute the basement of p73 function as a tissue architect. In a context dependent manner, TAp73 will combine the regulation of this basic transcriptional model with other tissue specific transcriptional profiles to orchestrate complex morphogenic processes like ciliogenesis and/or PCP (Figure 4). In turn, the coordinated orchestration of these processes (cell adhesion, cytoskeleton dynamic ciliogenesis and PCP) by p73 impinges on the cellular activities, leading to tissue and organ scale functionality of complex microenvironments such as neurogenic niche, the respiratory and reproductive epithelium and, maybe, the vascular network. Thus, based in the compiled data available on p73 physiological function, we propose that p73 might function as a tissue architect, and not just as another p53-Doppelgänger (Kaelin, 1998).

## Author Contributions

MCM and MMM conceived the review and took the lead in writing. LM-A performed data analysis and crafted the figures. LL-F performed a critical revision of the article. All authors provided critical feedback and contributed to the final manuscript.

## Conflict of Interest

The authors declare that the research was conducted in the absence of any commercial or financial relationships that could be construed as a potential conflict of interest.

## Publisher’s Note

All claims expressed in this article are solely those of the authors and do not necessarily represent those of their affiliated organizations, or those of the publisher, the editors and the reviewers. Any product that may be evaluated in this article, or claim that may be made by its manufacturer, is not guaranteed or endorsed by the publisher.
